# Pancreatic Endometriosis Coexisting with a Splenic Mesothelial Cyst: A Rare Case Report and Review of the Literature

**DOI:** 10.3390/diseases13070203

**Published:** 2025-06-30

**Authors:** Daniel Paramythiotis, Antonia Syrnioti, Dimitrios Tsavdaris, Aikaterini Smprini, Alexandros Mekras, Athanasios Apostolidis, Angeliki Cheva

**Affiliations:** 1 First Propaedeutic Surgery Department, University General Hospital of Thessaloniki AHEPA, Aristotle University of Thessaloniki, 54636 Thessaloniki, Greece; tsavdaris@auth.gr (D.T.); ksmprini@hotmail.com (A.S.); stlsa@auth.gr (A.A.); 2 Pathology Department, Faculty of Medicine, Aristotle University of Thessaloniki, 54124 Thessaloniki, Greece; asyrnioti@auth.gr (A.S.); antacheva@auth.gr (A.C.); 3 Department of General and Visceral Surgery, SHG-Klinikum Merzig, Academic Hospital of University of Saarland, 66663 Merzig, Germany; almekras@yahoo.gr

**Keywords:** pancreatic endometriosis, splenic mesothelial cyst, case report

## Abstract

Endometriosis is a clinical entity affecting up to 10% of women of reproductive age, characterized by ectopic endometrial tissue outside the uterine cavity. While extrapelvic endometriosis has been documented, pancreatic endometriosis remains extremely rare and poses significant diagnostic challenges due to its similarity to other pancreatic diseases. At the same time, splenic mesothelial cysts are also rare and typically benign. This report presents a unique case of pancreatic endometriosis coexisting with a splenic mesothelial cyst in a 31-year-old woman. The patient presented to the emergency department with complaints of persistent epigastric and low back pain. She noted having similar symptoms approximately a year prior. Her past medical history was otherwise unremarkable, and there was no known family history of pancreatic disease or neoplasms. Initial imaging revealed a 3.8 cm cystic lesion in the pancreatic tail, with features suggestive of mucinous cystadenoma. Following clinical evaluation and confirmation of the cyst’s nature through endoscopic ultrasound-guided biopsy, the patient subsequently underwent laparoscopic distal pancreatectomy and splenectomy due to worsening symptoms. Gross examination revealed a multilocular pancreatic cyst with a smooth, hemorrhagic wall. Microscopic analysis showed the cyst to be lined by cuboidal to columnar epithelium, consistent with pancreatic endometriosis, confirmed by immunohistochemical staining. The spleen showed cystic formations, diagnosed as a multifaceted mesothelial cyst. In conclusion, this report is the first to document the coexistence of pancreatic endometriosis and splenic mesothelial cysts, highlighting the importance of accurate imaging and pathologic evaluation in the diagnosis of these rare conditions. Early diagnosis and surgical intervention lead to favorable outcomes, reinforcing the importance of comprehensive diagnostic strategies.

## 1. Introduction

Endometriosis is generally common, with a prevalence ranging from 5 to 10% in women of reproductive age [1]. It is characterized by the presence of ectopic endometrial tissue outside the uterine cavity, affecting mainly the pelvic structures. The exact cause of endometriosis is not fully understood. Extra-pelvic endometriosis has also been well-documented and may involve several sites, including the gastrointestinal tract, abdominal wall, or thoracic cavity [2].

The occurrence of endometriotic lesions within the pancreas is exceedingly rare and poses a significant diagnostic challenge since it often mimics the more frequently encountered serous and mucinous cystic lesions of the pancreas [3]. This condition can cause symptoms such as abdominal pain, acute pancreatitis, weight loss, or even acute abdomen. Imaging tests such as computed tomography (CT), magnetic resonance imaging (MRI), and endoscopic ultrasound (EUS) can help, but definitive diagnosis often requires surgery. The prognosis for pancreatic endometriosis is generally good after surgical removal. However, early diagnosis and treatment are crucial to avoid complications such as chronic pancreatitis or cyst rupture [3,4].

At the same time, splenic mesothelial cysts are also rare benign cysts arising from the mesothelial cell lining of the splenic capsule. While their exact etiology remains unknown, they are generally thought to be developmental in origin. They are typically asymptomatic and discovered incidentally during imaging studies or surgical procedures. Radiologically, splenic mesothelial cysts are described as unilateral and smooth, with well-defined borders. Due to their rarity and benign nature, differentiation from other cystic lesions of the spleen is crucial for appropriate clinical management. Although their frequency is unknown, they occur more often in children and young adults [5,6].

In this report, we present a case of pancreatic endometriosis coexisting with a splenic mesothelial cyst in a 31-year-old female patient. To the best of our knowledge, this is the only case report documenting the coexistence of this condition. We aim to highlight the clinical, imaging, and pathological features of pancreatic endometriosis, with an emphasis on reviewing the available literature on this uncommon condition to provide insight into its diagnostic complexity and therapeutic approaches.

## 2. Case Report

A 31-year-old female presented with moderate epigastric and back pain that had persisted for several months. The pain exhibited periodicity but had no correlation with her menstrual cycle. Her past medical history was otherwise unremarkable. The patient had no history of smoking or alcohol consumption, no prior pancreatic disease, and was not taking any medications. Furthermore, there was no family history of pancreatic disease. On physical assessment, slight tenderness in the epigastric area was noted. Laboratory findings, including serum levels of tumor markers (CA19-9, CA125, and CEA), were within normal limits.

### 2.1. Imaging Findings

**Figure 1:**
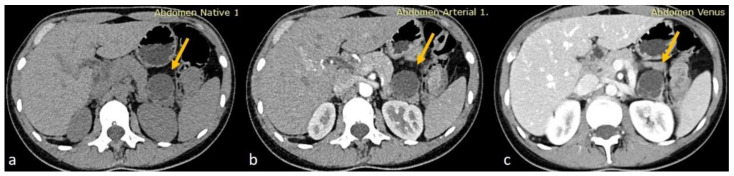
(**a**) Axial non-enhanced CT of the abdomen showing a cystic lesion at the tail of the pancreas with thin septations. No wall calcifications appreciated (yellow arrows). (**b**,**c**) Axial contrast-enhanced CT sections in arterial and venous phases, respectively, showing minimal wall enhancement of the cyst wall and the septations of the pancreatic cyst with no internal solid component.

Given the moderate severity of her pain, a CT scan of the upper and lower abdomen, along with the retroperitoneal space, was performed using a polytomous technique in both arterial and venous phases after the rapid administration of contrast. The scan revealed a 3.8 cm hypodense cystic lesion located in the tail of the pancreas. The lesion exerted pressure on the adjacent pancreatic tissue but showed no dilation of the main pancreatic duct. It had a smooth, mildly enhanced wall with internal septations, without any calcifications or solid components. A 9 mm hemangioma was also observed in the liver, and no other significant abnormalities were found in the kidneys, adrenal glands, uterus, or ovaries. The findings were consistent with a mucinous cystadenoma as the primary diagnosis, with other possible differentials including a pancreatic neuroendocrine tumor (PNET), simple cyst, or lymphangioma (Figure 1).

**Figure 2:**
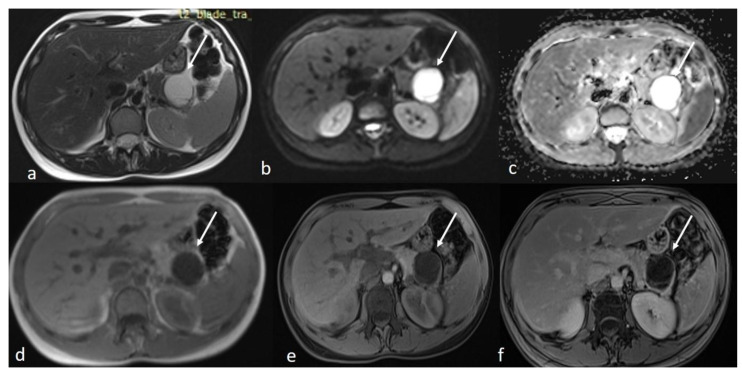
First MRI axial sequences of the upper abdomen. (**a**) T2 Blade axial sequence showing hyperintensity of the cyst (white arrows). (**b**,**c**) DWI and ADC showing no internal restriction of the cystic lesion. (**d**) T1 Vibe showing a hypointense cystic lesion at the tail of the pancreas. (**e**,**f**) T1 Vibe post-contrast at arterial and venous phases showing only minimal enhancement of the cyst wall and the septations with no internal solid component.

An MRI of the abdomen, performed approximately one year prior to admission, revealed a cystic thin-walled formation with internal septa, located in the dorsal part of the tail of the pancreas. The lesion measured approximately 3.9 cm in diameter and exerted pressure between the pancreas and the greater curvature of the stomach. The walls and internal diaphragms of the cyst showed enhancement post-contrast injection. There was no evidence of pancreatic duct dilation. Differential diagnoses at this stage included pancreatic cystic lesions such as cystadenoma, though the patient’s age was considered atypical, as well as other lesions like cystic lymphangioma or replication cysts. Additionally, incidental findings of mild hepatomegaly and a 9.5 mm hemangioma in the eighth hepatic segment were noted, along with small lymph nodes (Figure 2). In the absence of high-risk features or significant symptoms, a watch-and-wait approach with periodic monitoring was deemed appropriate.

An ultrasound of the liver, gallbladder, spleen, pancreas, kidneys, and ovaries, performed approximately three months after the MRI, revealed a lobular anechoic formation at the tail of the pancreas with a maximum size of 3.8 cm, further supporting the diagnosis of a pancreatic cyst. The liver, spleen, and other organs appeared normal, with the exception of small cystic formations in the ovaries and a moderate amount of pelvic fluid.

**Figure 3:**
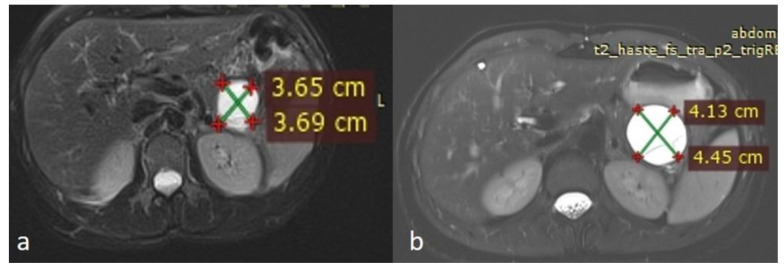
(**a**,**b**) Axial sequences of the upper abdomen comparison showed an increase in the lesion size.

**Figure 4:**
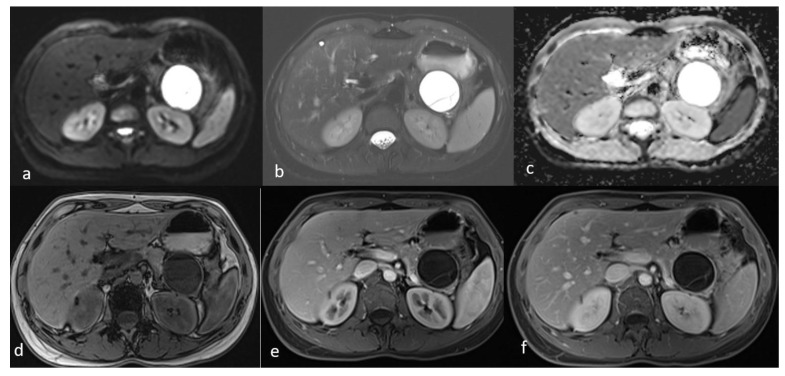
Second MRI follow-up after 6 months showed an increase in the lesion size. (**a**) T2 Blade axial sequence showing hyperintensity of the cyst with no internal solid component. (**b**,**c**) DWI and ADC again showing no internal restriction of the cystic lesion. (**d**) T1 Vibe showing a hypointense cystic lesion at the tail of the pancreas. (**e**,**f**) T1 Vibe post-contrast at arterial and venous phases showing no change in the enhancement of the cyst.

An MRI performed one year after the initial imaging and two months before admission revealed an increase in the size of the pancreatic cystic lesion to 4.5 cm (Figure 3 and Figure 4) with enhancement of its thin wall and internal septa, confirming its progressive nature. The lesion continued to compress the pancreatic tail and the greater curvature of the stomach, without causing pancreatic duct dilation. The imaging also showed low signal intensity on T1- and high signal intensity on T2-weighted sequences, with capsule enhancement post-contrast, suggesting a mucinous cystic neoplasm (Figure 4). Additionally, the MRI confirmed the presence of a 9 mm hemangioma and a small liver cyst, both unchanged from prior scans. No enlarged retroperitoneal lymph nodes were observed, and the MRCP findings showed normal intrahepatic bile ducts, common hepatic bile duct, gallbladder, and pancreatic duct. No abnormalities were found in the spleen, kidneys, or adrenal glands.

### 2.2. Surgical Procedure and Postoperative Course

**Figure 5:**
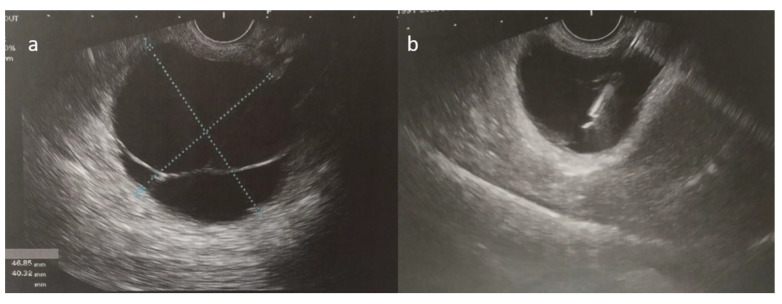
(**a**,**b**) Ultrasound-guided fine-needle aspiration of the cystic fluid was performed.

As her symptoms worsened over time, the patient ultimately presented to the emergency department with persistent epigastric and lower back pain. Following a complete clinical, laboratory, and imaging workup, the pancreatic cyst was confirmed through an ultrasound-guided fine-needle aspiration, which suggested a mucinous cystic neoplasm with no signs of dysplasia (Figure 5). The patient then underwent a laparoscopic distal pancreatectomy and splenectomy. Initially, the surgery was uneventful, and the patient was extubated and monitored in the ICU for one day before being transferred to the surgical ward.

Two days post-surgery, shortly after her transfer from the ICU to the surgical ward, the patient experienced a drop in hemoglobin levels, raising concerns about potential intra-abdominal bleeding. She was taken back to the operating room for laparoscopic exploration; however, no active source of bleeding was identified. Postoperatively, her hemoglobin stabilized with supportive care, including the administration of two units of packed red blood cells. Following this, she demonstrated steady clinical improvement, mobilized without difficulty, and was discharged in a stable condition on the eighth postoperative day. Post-surgical instructions included wound care, anticoagulation therapy (tinzaparin 0.45 mL for 20 days), and a vaccination schedule (pneumococcal, H. influenzae, and meningococcal vaccines), without antibiotic treatment. Her biopsy results were scheduled for review 20 days later. At her one-year follow-up, the patient remained asymptomatic, with no evidence of recurrence on imaging or laboratory evaluation, indicating a favorable long-term outcome.

### 2.3. Pathological Findings

Gross examination of the distal pancreatectomy with splenectomy specimen revealed a 4.5 × 4.2 × 4.1 multiloculated cyst at the dorsal region of the pancreatic tail, with a smooth, often hemorrhagic wall. In addition, at the surface of the spleen, multiple microscopic protrusions of cystic composition were identified, measuring from 0.4 to 0.9 cm in diameter.

**Figure 6:**
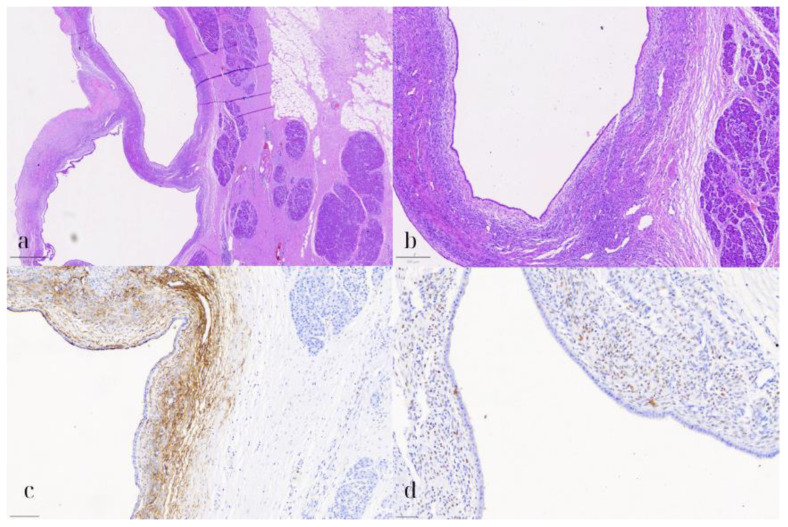
Pancreatic endometriotic cyst. (**a**) HE1: Hematoxylin and eosin stain (HE) showing the cyst lined by cuboidal to low columnar epithelium with underlying spindle cell stroma (800 μm). (**b**) HE2: Higher magnification (HE) highlighting the lack of nuclear pleomorphism, atypia, or mitotic activity in both the epithelial and stromal cells (200 μm). (**c**) CD10: Immunohistochemical staining positive for CD10, marking the stromal cells consistent with endometrial stroma (100 μm). (**d**) ER: Positive estrogen receptor (ER) expression in the stromal cells, supporting the diagnosis of a pancreatic endometriotic cyst (50 μm).

On microscopic examination, the pancreatic cyst was lined by a single layer of cuboidal to low columnar, occasionally ciliated, epithelium (Figure 6). Underneath the epithelium, a thin layer of stroma was observed, consisting of spindle cells, arranged in loosely interconnected bundles. Both the epithelial and stromal cells demonstrated no significant nuclear pleomorphism, atypia, or mitotic activity. On immunohistochemical evaluation, the epithelial cells were positive for CK7, while the stromal cells expressed CD10, WT1, and estrogen receptor (ER). The pancreatic parenchyma was otherwise unremarkable. No evidence of intraductal papillary mucinous neoplasm (IPMN) was present. These findings were consistent with a pancreatic endometriotic cyst.

**Figure 7:**
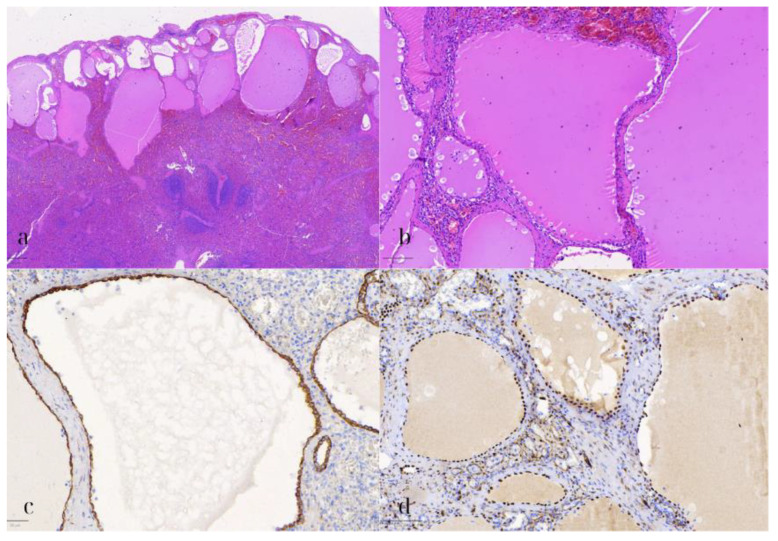
Splenic mesothelial cyst. (**a**) HE1: Hematoxylin and eosin stain (HE) of the splenic cyst lined by flat to cuboidal mesothelial cells, with eosinophilic cytoplasm and elongated nuclei (500 μm). (**b**) HE2: Higher magnification (HE) showing the benign appearance of the mesothelial cells, without significant nuclear pleomorphism or mitotic activity (100 μm). (**c**) CK8/18: Positive immunohistochemical staining for CK8/18 in the mesothelial cells, indicating epithelial origin (50 μm). (**d**) WT1: WT1-positive staining in the mesothelial cells, further confirming the diagnosis of a splenic mesothelial cyst (100 μm).

Additionally, microscopic examination of the spleen demonstrated several cystic formations of varying size and shape, with a distinct subcapsular localization (Figure 7). These were lined by a single layer of flat to cuboidal cells, with eosinophilic cytoplasm, and elongated to ovoid nuclei, lacking any significant nuclear pleomorphism, atypia, or mitotic activity. Upon immunohistochemical assessment, these cells exhibited the following immunophenotype: CK8/18+, WT1+, D2-40+, CD31-, ERG-, and CD68-. The immunohistochemical stain for CD68 also highlighted the abundant histiocytes surrounding the cystic formations. The adjacent splenic parenchyma did not demonstrate any remarkable findings. As a result, the diagnosis of a multilocular splenic mesothelial cyst was established.

## 3. Discussion

This case highlights the diagnostic complexity and rarity of both pancreatic endometriosis and splenic mesothelial cysts. To our knowledge, this is the only available case report documenting the coexistence of pancreatic endometriosis and splenic mesothelial cysts. Each entity, while uncommon, presents unique challenges, especially when attempting to distinguish them from more common cystic lesions in these organs.

Endometriosis outside of the pelvic region, particularly within the pancreas, is extremely rare [2]. A high index of clinical suspicion is essential in premenopausal or perimenopausal women presenting with pancreatic cysts, especially those located in the body or tail, who report cyclical abdominal pain correlated with their menstrual cycle or have a known history of endometriosis [7,8]. MRI findings can be informative, since pancreatic endometriotic cysts may show hyperintensity on T1-weighted images during episodes of bleeding, while they typically appear hypointense on T1-weighted and T2-weighted images when hemorrhage is absent [8,9]. Moreover, on EUS, cystic fluid with hemorrhagic content may be detected [8]. Nevertheless, definitive diagnosis remains challenging due to the overlapping imaging features with other, more common pancreatic cystic lesions, including mucinous cystic neoplasms and intraductal papillary mucinous neoplasms (IPMN), both of which carry significant risk for malignancy [3,9,10]. Therefore, diagnosis frequently relies on histopathological assessment of the resected specimen [4]. In this case, the loculated cyst in the pancreatic tail was initially suspected to be a mucinous neoplasm. Nevertheless, microscopic examination revealed the presence of endometrial-type epithelium and stroma. The latter, on immunohistochemical examination, stained positive for CD10 and estrogen receptor (ER), confirming the diagnosis of endometriosis.

Although pancreatic endometriosis is benign, its presentation can mimic neoplastic conditions and may result in persistent abdominal or back pain due to mass effect or hemorrhage into the cyst. If a preoperative diagnosis is established, surgical resection may be unnecessary, with the primary treatment involving the suppression of ovarian function [8,11]. However, in situations where the cyst increases in size, pain management is ineffective, or there is a concern for malignancy, surgical resection may be warranted [3,4,11].

Splenic mesothelial cysts are exceedingly rare, comprising only a small fraction of non-parasitic splenic cysts [6]. Their etiology remains uncertain, with prevailing theories suggesting a developmental origin [12]. Due to their rarity, the preoperative differentiation of splenic mesothelial cysts from other cystic lesions, such as epidermoid cysts or pseudocysts, is challenging, with imaging studies often proving inconclusive [6]. In the current case, histopathological analysis revealed multilocular cysts lined by flat to cuboidal mesothelial cells, which stained positive for CK8/18, WT1, and D2-40, confirming the diagnosis. Although benign and often asymptomatic, large or symptomatic mesothelial cysts may require surgical intervention due to the risk of complications such as rupture or hemorrhage [13]. In this case, the patient underwent a splenectomy, allowing for the complete resection of the cysts and the prevention of potential future complications.

The coexistence of pancreatic endometriosis with a splenic mesothelial cyst is exceptionally rare, but several pathophysiological and molecular mechanisms may underlie their simultaneous development. Both lesions are thought to arise from the peritoneal mesothelium, which is capable of undergoing metaplastic transformation under chronic inflammatory or hormonal influences. Coelomic metaplasia, a process in which mesothelial cells transform into endometrial-like or cystic mesothelial tissue, is a central mechanism implicated in both entities [14].

Endometriosis outside the pelvis, such as in the pancreas, is believed to result from lymphatic or hematogenous dissemination of endometrial cells, or from in situ coelomic metaplasia of the peritoneal lining, including the peripancreatic and splenic surfaces [7,14]. The local inflammatory environment created by ectopic endometrial tissue can promote mesothelial proliferation and cyst formation, as chronic inflammation and adhesions are known to induce reactive mesothelial hyperplasia and cystic change [15,16]. Molecularly, this process is driven by proinflammatory cytokines (e.g., TNF-α, IL-1β), growth factors (e.g., VEGF, IGF), and altered expression of adhesion molecules (e.g., integrins, ICAM), which facilitate both the implantation of endometrial cells and the proliferation of mesothelial cells [14].

Histological studies have demonstrated that endometriosis can directly contribute to the development of multicystic mesothelial lesions, supporting a secondary, non-neoplastic origin for some mesothelial cysts in the context of endometriosis [15]. Thus, the simultaneous occurrence of these lesions likely reflects a shared pathogenesis involving chronic inflammation, hormonal stimulation, and mesothelial plasticity in the peritoneal environment [14]. Surgical intervention is typically indicated when pancreatic endometriosis coexists with a splenic mesothelial cyst because preoperative distinction between benign, premalignant, and malignant pancreatic cystic lesions is highly challenging. Imaging and cyst fluid analysis often cannot reliably differentiate endometriosis or mesothelial cysts from mucinous cystic neoplasms or other cystic tumors with malignant potential, leading to a significant risk of misdiagnosis if managed non-operatively. The American College of Gastroenterology recommends surgical resection for pancreatic cysts when malignancy cannot be excluded, when the cyst is symptomatic, or when there are high-risk features such as size, mural nodules, or main duct involvement [17].

A literature review was conducted using MedLine and Scopus databases, focusing on cases of pancreatic endometriosis. This search yielded only eighteen case reports, of which one was missing the main text, and another was available solely in Spanish. The remaining sixteen case reports [3,7,8,9,10,18,19,20,21,22,23,24,25,26,27,28] were published from 1984 to 2024, and from them were drawn the clinical manifestations, diagnostic challenges, and treatment outcomes associated with pancreatic endometriosis, a rare entity often misdiagnosed due to its overlap with other pancreatic cystic lesions. As for the ages of the patients, it seems to range from their 20s to early 50s, with a median age of around 35–40 years. Epigastric pain was the most frequently reported symptom, while some patients reported that the pain radiated to the back or chest. A few cases also presented with weight loss, nausea, vomiting, diarrhea, or anorexia. Seven patients actually reported a correlation between pain and the menstrual cycle, suggesting a possible hormonal influence. Most lesions were located in the pancreatic tail or body, with cyst sizes ranging from 2.2 cm to as large as 16 cm. Larger cysts were more likely to raise suspicion of a malignant lesion or cystic neoplasm. Pancreatic endometriosis was frequently misdiagnosed as mucinous cystic adenoma or pseudocyst due to similarities in imaging findings. Some cases were also thought to be malignant or premalignant lesions. Most patients underwent distal pancreatectomy (PE), often with splenectomy (SE). In some cases, laparoscopic or robotic techniques were used. The majority of patients had uneventful recoveries, although minor complications like respiratory issues or pain were reported in a few cases. Notably, there were no long-term recurrences of pancreatic endometriosis, and most patients remained asymptomatic at follow-up. These findings are summarized in Table 1.

Regarding the pre-operative assessment of the patients, this was mainly based on CT and MRI imaging examinations, as well as on blood tests. The findings are summarized in Table 2. Many studies reported cystic masses in various locations within the pancreas. Marchevsky et al. [19] noted a cystic mass in the tail, while Tunuguntla et al. [18] observed a cystic mass in the tail abutting the spleen, with follow-up CT showing reaccumulation. Lee et al. [26] described an oval-shaped low-density cystic mass with distinct capsular enhancement and suspicious internal septations. On the contrary, studies such as those by Oishi et al. [8] and Karaosmanoglu et al. [23] identified cystic lesions with specific features like thick walls and mural nodules, indicating the complexity of these lesions. Typically, MRI findings were consistent with cystic structures, some showing features like minimal wall enhancement, hyperintensity on T2-weighted images, and no evidence of main duct dilation.

Laboratory tests showed a range of results. Elevated levels of amylase were noted in studies by Marchevsky et al. [19], Tunuguntla et al. [18], and Karaosmanoglu et al. [23], indicating pancreatic involvement. Additionally, other studies reported elevated levels of CEA (carcinoembryonic antigen) (Mederos et al. [25] and AlNuaimi et al. [9]), suggesting the potential for malignancy or inflammation. Normal laboratory results were also noted in five studies [3,8,21,24,28], indicating that not all cases of pancreatic endometriosis present with elevated tumor markers or other laboratory abnormalities.

Over the past four decades, the evaluation of pancreatic cystic lesions has progressed from rudimentary CT-based detection and elementary enzymatic assays to comprehensive, multimodal diagnostic paradigms. In the 1980s and early 1990s, conventional CT imaging afforded only gross localization of hypodense cystic collections, while laboratory investigations were largely confined to serum amylase determinations and occasional tumor marker assessments, data that seldom influenced therapeutic decision making. The advent of multi-detector CT at the turn of the millennium permitted enhanced delineation of cystic morphology, including capsular enhancement, septal architecture, and evidence of fluid reaccumulation; nonetheless, MRI remained an underutilized adjunct despite its superior soft-tissue contrast and functional imaging capabilities.

By the mid-2010s, MRI had achieved parity with high-resolution CT, leveraging T1- and T2-weighted sequences, diffusion-weighted protocols, and dynamic contrast studies to more accurately characterize intracystic contents, wall thickness, and hemorrhagic or proteinaceous components. Laboratory panels also broadened to incorporate inflammatory biomarkers (e.g., CRP, γ-GT) and cyst fluid tumor antigens (e.g., CEA), demonstrating an awareness of the interplay between inflammatory, hemorrhagic, and neoplastic processes. Contemporary protocols, including submillimeter CT reconstructions, MR cholangiopancreatography, quantitative diffusion measurements, and detailed biochemical profiling, permit elegant risk stratification, thereby simplifying the equilibrium between surveillance and surgery for lesions with a risk of malignancy.

The diagnostic pathway in earlier eras was further constrained by the absence of EUS-FNA and limited immunohistochemical panels. Definitive diagnosis was almost invariably made post-resection through histopathology, often with only ER/PR staining or basic cytokeratins, without markers such as CD10, which are now routinely employed to identify endometrial stroma or mesothelial cells. These advances not only validate the clinical and radiographic patterns first recognized decades ago but also markedly reduce unnecessary surgery and improve patient counseling and outcomes.

Finally, the pathological findings of these studies were collected and distinguished into gross surgical findings, microscopic findings, and immunohistochemical findings, and are summarized in Table 3. Notably, the gross findings indicate significant variability, from well-circumscribed cysts with smooth walls to more complex multicystic lesions exhibiting hemorrhage and fibrosis. These observations highlight the morphologic diversity of pancreatic endometriosis, indicating that a broad differential diagnosis should be maintained when evaluating pancreatic cystic lesions.

Microscopically, most studies report the presence of endometrial glands and stroma, often accompanied by hemosiderin deposition and macrophage infiltration. This finding emphasizes that the histopathological features of pancreatic endometriosis may mimic those of ovarian endometriomas, complicating the diagnostic process. The identification of hemosiderin-laden macrophages in several studies indicates recurrent bleeding, a feature of endometriosis, and highlights the potential for chronicity and complications such as fibrosis and scarring of the affected pancreatic tissue.

The immunohistochemical analyses from the studies on pancreatic endometriosis reveal several key findings. ERs were reported to be positive in multiple studies, indicating the hormonal influence on the lesions, particularly in the studies of Lee et al., Yamamoto et al., and Karaosmanoglu et al. [7,23,26]. Additionally, positivity for PR was observed in some cases, further supporting the role of hormones in the pathology, as noted in the studies by Lee et al., Yamamoto et al., and AlNuaimi et al. [7,9,26].

CD10 was consistently positive across several studies, highlighting the presence of endometrial stroma and aiding in diagnosis, particularly in the reports of Oishi et al., Monrad-Hansen et al., and AlNuaimi et al. [8,9,28]. Cytokeratin markers CK-7 and CK-19 were also noted to be positive in one of the studies, suggesting epithelial differentiation, as reported by AlNuaimi et al. [9]. Furthermore, positive expression of CA19-9 was documented in the study by Verbeke et al., indicating potential utility in distinguishing endometriotic tissue [24].

This case of synchronous pancreatic endometriosis and splenic mesothelial cysts carries several important clinical implications. First, it underscores the necessity of including endometriosis in the differential diagnosis of cystic pancreatic lesions—particularly in reproductive-aged women with cyclic or unexplained epigastric pain—to avoid misclassification as mucinous neoplasms or pseudocysts and thereby reduce unnecessary major pancreatic resections. Second, it highlights the value of multiparametric MRI and EUS-FNA with comprehensive immunohistochemical panels (including CD10, ER, WT1, and D2-40) in distinguishing hormonally driven, hemorrhagic endometrial implants and reactive mesothelial cysts from potentially malignant entities. Third, although rare, peritoneal mesothelial hyperplasia and cystic lesions can occur in association with endometriosis, typically as localized reactive processes. In the presence of atypical or multifocal cystic findings, careful assessment of the involved areas is warranted; however, routine extensive intra-abdominal exploration beyond the pancreas is not justified unless supported by specific clinical indications. Finally, this case reinforces the benefit of a multidisciplinary approach, integrating surgical, radiologic, pathologic, and gynecologic expertise to optimize diagnostic accuracy, preserve organ function, and tailor individualized management plans.

Future investigations should aim to establish the true prevalence and natural history of extra-pelvic endometriotic and mesothelial lesions through multicenter registries that collect standardized imaging, histopathologic, and clinical outcome data. Prospective evaluation needs to be performed to assess the performance parameters of advanced MRI sequences (e.g., diffusion-weighted imaging, dynamic contrast enhancement) and new molecular markers (e.g., microRNA profiles, circulating cell-free DNA) in noninvasively characterizing these rare cystic lesions. Experimental studies of targeted medical treatments, such as selective aromatase inhibitors, matrix metalloproteinase antagonists, or anti-VEGF agents, potentially would offer non-surgical treatments, especially for multifocal or high-risk patients. Finally, mechanistic studies using in vitro models of mesothelial metaplasia and co-culture systems of endometrial and mesothelial cells may elucidate the molecular crosstalk involved in lesion initiation and development, which may reveal novel biomarkers and therapeutic targets.

## 4. Conclusions

This case underscores that pancreatic endometriosis, while rare, should be a differential consideration in reproductive-age women presenting with cystic pancreatic lesions, particularly when imaging suggests mucinous neoplasm but lacks high-risk features. It highlights the limitations of imaging and cytology in distinguishing benign from malignant lesions, reinforcing the need for histopathological and immunohistochemical analysis for definitive diagnosis. Awareness of this entity is crucial, as misdiagnosis can lead to overtreatment, including unnecessary pancreatectomy or splenectomy. This report expands the clinical spectrum of extrapelvic endometriosis and stresses the importance of integrating clinical, radiologic, and pathologic data to avoid mismanagement. Moreover, the coexistence of pancreatic endometriosis with a splenic mesothelial cyst, though exceptionally rare, suggests a shared pathogenesis rooted in peritoneal mesothelial plasticity. Chronic inflammatory or hormonal stimuli may induce coelomic metaplasia, leading to the development of both endometrial-like and cystic mesothelial lesions. This association highlights the role of the inflammatory microenvironment and underscores the importance of considering multifocal reactive processes in atypical cystic presentations. When preoperative distinction from neoplastic lesions is uncertain, surgical intervention remains justified.

## Figures and Tables

**Table 1:** Baseline characteristics of studies reporting cases of pancreatic endometriosis. Abbreviations: Intraductal Papillary Mucinous Neoplasm (IPMN), Pancreatectomy (PE), Splenectomy (SE), Gastrectomy (GE).

Study ID	Patient Demographics (Age)	Clinical Presentation	Emergency Presentation	Menstrual History	Anatomic Location	Diameter (cm)	Preoperative Diagnosis	**Treatment**	**Postoperative Course**
Marchevsky et al., 1984 [19]	36	Epigastric pain radiating to back	No	Normal	Tail	4.5	Endometriosis	Distal PE and SE, and cholecystectomy	Uneventful
Goswami et al., 1985 [20]	40	Left flank pain	No	Normal	Tail	8	Malignant lesion	Distal PE and SE along with left radical nephrectomy	-
Verbeke et al., 1996 [24]	28	Epigastric pain	No	Pain correlated with menstrual cycle	Body and Tail	4	Postinflammatory pseudocyst	PE and SE	Uneventful
Lee et al., 2002 [26]	21	Epigastric pain Weight loss	No	Normal	Body	4	Mucinous cystic adenoma	Partial PE	Uneventful
Tunuguntla et al., 2004 [18]	34	Acute epigastric pain	Yes	Normal	Tail	8	Suspected cystic neoplasm	Distal PE and SE	Uneventful
Oishi et al., 2011 [8]	35	Epigastric pain radiating to back and chest	No	Pain correlated with menstrual cycle	Body	4	Mucinous cystic adenoma	Distal PE and SE	Severe pain on postoperative day 1
Monrad-Hansen et al., 2012 [28]	43	Acute epigastric pain	Yes	Irregular cycle	-	-	Suspected endometriosis and carcinomatosis	Explorative laparoscopy, left oophorectomy, drainage of cyst.	Uneventful
Assifi et al., 2014 [10]	32	Abdominal pain	-	-	Tail	-	-	Distal PE and SE	-
	63	None	-	-	Body	-	-	Distal PE and SE	-
Plodeck et al., 2016 [27]	72	Abdominal pain	No	Hysterectomy	Tail	2.2	Mucinous cystic adenoma	Left hemipancreatectomy	Minor respiratory issues
Mederos et al., 2017 [25]	43	Abdominal pain, fatigue, diarrhea, anorexia, weight loss	No	Normal	Body and Tail	16	Premalignant pancreatic cystic lesion	Laparoscopic distal PE and SE	Uneventful
Yamamoto et al., 2018 [7]	26	Acute epigastric pain	Yes	Irregular cycle	Body	7	Mucinous cystic adenoma	Laparoscopic distal PE and SE	Uneventful
Karaosmanoglu et al., 2020 [23]	38	Acute epigastric pain	Yes	Normal	Tail	10	Complicated acute pancreatitis or ruptured mucinous cystic adenoma	Distal PE, subtotal distal GE, and SE	Uneventful
Huang et al., 2021 [3]	51	Epigastric pain radiating to back, diarrhea with greasy stools, and anorexia without weight loss	No	Hysterectomy	Body and Tail	3.7	IPMN	Robotic distal PE and splenectomy	Uneventful
Ludwig et al., 2021 [22]	Early 30s	Acute abdominal pain	Yes	Appendiceal endometriosis	Body	11	Mesenteric duplication cyst, pancreatic pseudocyst, and endometrioma	Robotic potentially pancreas-sparing cyst resection with possible cystogastrostomy	Recurrent epigastric pain
AlNuaimi et al., 2023 [9]	31	Epigastric pain, nausea, vomiting	Yes	Normal	Tail	3	Mucinous cystic adenoma	Distal subtotal robotic PE	Uneventful
Gutiérrez et al., 2024 [21]	29	Epigastric pain, nausea, vomiting, dysphagia, and weight loss	Yes	Normal	Body	15	Malignant or premalignant lesion	Exploratory laparotomy	Uneventful

**Table 2:** Pre-operative assessment of the patients. Abbreviations: Cancer Antigen 19-9 (CA 19-9), Carcinoembryonic Antigen (CEA), White Blood Cells (WBC).

Study ID	CT	MRI	**Laboratory**
Marchevsky et al., 1984 [19]	Cystic mass in the tail of the pancreas.	-	Elevated amylase
Goswami et al., 1985 [20]	-	-	-
Verbeke et al., 1996 [24]	-	-	Normal
Lee et al., 2002 [26]	Oval-shaped low-density cystic mass in the pancreatic body with bulging-out contour, distinct capsular enhancement, and suspicious internal septations.	-	Normal (CA 19-9 mildly elevated)
Tunuguntla et al., 2004 [18]	Cystic mass in the tail of the pancreas abutting the spleen. Follow-up CT showed reaccumulation of the cystic collection.	-	Elevated amylase
Oishi et al., 2011 [8]	Cystic lesion in pancreatic body.	Multilocular cyst with septal wall, no ductal dilatation or stenosis.	Normal
Monrad-Hansen et al., 2012 [28]	Peripancreatic swelling, tissue changes around the pancreas and mesentery	Hyperintense T1 and hypointense T2 signals around pancreas, consistent with blood products, no diffusion restriction or contrast enhancement.	Normal
Plodeck et al., 2016 [27]	Partly cystic, partly solid lesion in pancreatic tail with small calcification.	Partially cystic, partially hemorrhagic lesion with subtle rim enhancement, pancreatic tail hypotrophy.	Elevated GGT, ALAT, CRP
Mederos et al., 2017 [25]	Macrocystic, well-circumscribed mass with internal septations, no nodules or internal enhancement.	-	Elevated cystic CEA
Yamamoto et al., 2018 [7]	Cystic lesion in pancreatic body.	Cystic lesion, no ductal dilatation, stenosis, or displacement.	Elevated WBC
Karaosmanoglu et al., 2020 [23]	Cystic mass with thick walls, mural papillary nodules, invading splenic parenchyma.	Confirmed CT findings, subcapsular fluid collection.	Elevated pancreatic amylase
Huang et al., 2021 [3]	-	Cystic lesion in the pancreatic body, consistent with IPMN, no main duct dilation or nodules.	Normal
Ludwig et al., 2021 [22]	Simple cystic mass in the left upper abdomen, medial to the lesser curvature of the stomach, inseparable from the anterior aspect of the pancreas.	Confirmed cyst with mass effect on pancreas and stomach.	-
AlNuaimi et al., 2023 [9]	Unilocular cyst in pancreatic tail, slight atrophy of the distal tail, no signs of acute pancreatitis.	Hypointense on T1, hyperintense on T2, minimal wall contrast enhancement, no diffusion restriction.	Elevated CEA and amylase
Gutiérrez et al., 2024 [21]	Poorly evaluable nodular appearance of the pancreatic gland. Cyst-like lesion in the body of the pancreas.	-	Normal

**Table 3:** Gross surgical findings, microscopic findings, and immunohistochemical findings of the included studies. Abbreviations: Estrogen receptor (ER), Progesterone receptor (PR).

Study ID	Gross Surgical Findings	Microscopic Findings	**Immunohistochemistry**
Marchevsky et al., 1984 [19]	Cyst, well-circumscribed, with a smooth, trabeculated, gray-brown wall and areas of hemorrhage.	Lined by fibrous tissue with endometrial glands (cuboidal and columnar cells) and a well-vascularized spindle cell stroma, hemosiderin deposition, and focal hemorrhage	-
Goswami et al., 1985 [20]	Compressed the posteroinferior aspect of the kidney, inner surface of the cyst smooth and dark brown in color. Compressed kidney was small and atrophic. Another smaller cyst compressing the pancreas with fibrotic tail.	Pancreatic tissue showed typical endometrial glands and stroma. Pancreatic and peripancreatic tissue showed considerable fibrosis and hemosiderin accumulation. Thick fibrous wall with numerous macrophages and abundant hemosiderin pigment, suggesting an endometrial cyst. Pyelitis and focal tubulointerstitial nephritis noted	-
Verbeke et al., 1996 [24]	Tumor with multiple cysts, walls of cysts grayish-white, smooth, brown-spotted surface.	Endometrial-type stroma, spindle cells, hemorrhage, hemosiderin-laden macrophages, varied epithelial cell types, slight fibrosis in pancreatic tissue	Positive for pancytokeratin, CA19-9; weak expression of CA125; negative for ER, PR, vimentin.
Lee et al. 2002 [26]	Well-circumscribed cyst in pancreatic stroma, gray-brown wall with focal hemorrhage.	Endometrial glands with spindle cell stroma, focal hemorrhage, and hemosiderin-laden macrophages	Positive for ER and nuclear PR.
Tunuguntla et al., 2004 [18]	Cyst in the tail of the pancreas; suspicious lesions on the right diaphragm and liver, and potential neoplastic areas on the spleen.	Atypical glandular structure	-
Oishi et al., 2011 [8]	Cyst, irregular inner surface, brownish-yellow and reddish-brown color, no pancreatic duct fistula.	Thick fibrous wall, endometrial glands with spindle cell stroma, focal hemorrhage, hemosiderin deposition	Positive for CD10, PR, negative for ER.
Monrad-Hansen et al., 2012 [28]	Cystic structure with liquified gray material (old, resorbed blood).	Peripancreatic fatty tissue with focal fibrosis, hemosiderin-laden macrophages	Stromal cells positive for CD10, indicative of endometriosis.
Plodeck et al., 2016 [27]	Multicystic lesion with hemorrhage and fibrosis.	Cysts lined with flat to cubic epithelium, ovarian-type stroma, significant hemorrhage, and dystrophic calcification	Smooth muscle cells visible, part of endometrial stroma.
Mederos et al., 2017 [25]	Unilocular cyst, gray-green cloudy fluid, smooth trabeculated gray-brown wall, focal hemorrhage.	Benign cubo-columnar epithelial lining, bland spindle cells, thin-walled blood vessels, hemosiderin-laden macrophages	Positive for CD10 and ER, negative for inhibin.
Yamamoto et al., 2018 [7]	Cyst with smooth, trabeculated gray-brown wall, focal hemorrhage.	Elongated endometrial glands with spindle cell stroma, areas of focal hemorrhage, hemosiderin-laden macrophages	Positive for CD10, ER, and PR; negative for inhibin.
Karaosmanoglu et al., 2020 [23]	Hemorrhagic cystic lesion with mural discontinuity, no clear separation between pancreatic tail and splenic hilum.	Glandular structures in endometrial background	Positive for ER in stromal cells.
Huang et al., 2021 [3]	Endometriotic cyst.	Endometrial stroma with hemosiderin-laden macrophages; no mucinous epithelium or ovarian-type stroma	-
Ludwig et al., 2021 [22]	Fibrinopurulent exudate, granulation tissue, fibrotic scar with eosinophils and plasma cells, indicating cyst infection and abscess.	Confirmed cyst with mass effect on pancreas and stomach	CD10 staining for endometrial stroma in the initial cyst.
AlNuaimi et al., 2023 [9]	Unilocular cyst lined by tall, non-mucin-producing cells with ovarian-type stromal component, inflammatory infiltrate, hemosiderin-laden macrophages.	Non-mucinous columnar cells, ovarian-type stroma, foamy histiocytes with hemosiderin	Positive for CD10, ER, CK-7, and CK-19.
Gutiérrez et al., 2024 [21]	Macroscopically identified as an endometrioma. Cystic liquid sampled for analysis.	Loose connective tissue and fibrosis zones with venules and mononuclear inflammatory cell infiltrate, including gold pigmentation attached to fibrous connective tissue, in addition to phagocytosis of hemosiderin by macrophage	-

## Data Availability

The original contributions presented in this study are included in the article. Further inquiries can be directed to the corresponding author.
